# Mitochondrial mass and low mitochondrial membrane potential percentage of CD8+ T cell subsets are implicated with therapeutic effect in depressive disorder

**DOI:** 10.3389/fpsyt.2025.1550958

**Published:** 2025-07-18

**Authors:** Yiran Huang, Zhenni Chen, Yang Xu, Biqiong Ren

**Affiliations:** ^1^ Clinical Medical College, Hunan University of Traditional Chinese Medicine, Changsha, China; ^2^ Department of Laboratory Medicine, The Second Xiangya Hospital of Central South University, Changsha, China; ^3^ Department of Science and Education, The Second People's Hospital of Hunan, Changsha, China

**Keywords:** depression, T lymphocytes, mitochondrial mass, low mitochondrial membrane potential, chronic low-grade inflammatory response

## Abstract

**Background:**

Depression involves immune-inflammatory responses and mitochondrial dysfunction in its pathophysiology. However, the association between mitochondrial alterations in T lymphocytes and clinical treatment responses in depression remains unclear.

**Methods:**

Forty hospitalized patients diagnosed with depression participated in this study, and peripheral blood samples were collected upon admission and discharge. Flow cytometry was used to monitor the frequency of T-lymphocyte subpopulations in depressed patients, mitochondrial mass (MM) and low mitochondrial membrane potential (MMP^Low^, %) was calculated by using the lymphocyte mitochondrial function analysis software (patented technology) in conjunction with the median fluorescence intensity of each subpopulation. Patients were further stratified into routine-term hospitalization (≤21 days) and long-term hospitalization (>21 days) groups to explore potential associations between hospitalization duration and mitochondrial changes.

**Results:**

The proportions of CD4+ and CD8+ T-cell subsets remained stable before and after treatment, while absolute counts of CD4+ central memory T (Tcm), CD4+ effector memory T (Tem) and CD8+ Tem significantly declined (*P* < 0.05). In terms of mitochondrial function, MM was significantly increased in CD4+ Tcm, CD8+ naïve T (Tn), CD8+ Tcm, CD8+ effector T (Tef), and CD8+ Tem subsets after treatment (*P* < 0.05), with no significant changes in CD4+ T subsets. Correspondingly, MMP^Low^ significantly decreased in CD4+ Tn, CD8+ Tn, CD8+ Tcm, and CD8+ Tem cells (*P* < 0.05), suggesting improved mitochondrial polarization. Exploratory subgroup analysis based on hospitalization duration revealed that these mitochondrial improvements were predominantly observed in the routine-term hospitalization group, whereas no significant changes were detected in the long-term hospitalization group.

**Conclusion:**

Our results suggest that mitochondrial alterations in CD8+ T-cell subsets may represent immunometabolic adaptations accompanying clinical improvement in depression. MM and MMP^Low^ in CD8+ T lymphocytes may serve as preliminary biomarkers for assessing therapeutic response in depression.

## 1 Introduction

Depression is a pervasive mental disorder that possesses attributes such as loss of interest, negative rumination, and fatigue, and has the severity of high morbidity, mortality, and disability (1), and is a major public health problem that is gaining prominence and posing a significant burden to the world (2). The pathogenesis of depression is unknown, and current views focus on monoamine neurotransmitter disorders, hypothalamus-pituitary-adrenal axis dysfunction, neurotrophic factor deficiencies, immune-inflammatory responses, and gut microbial disorders (3, 4). Despite extensive research in this area, the exact etiology of complex mental disorders has not been elucidated. Increasing evidence suggests that immune-inflammatory responses play a key role in the development of depression (5–7). T lymphocytes have received widespread attention for their functions in maintaining organismal immune homeostasis and modulating inflammatory responses. Depression patients have an abnormal T-cell subset composition, which is characterized by a skewing of T-cells towards effector or terminal differentiation and a reduction in regulatory T-cells (Tregs), suggesting an immune senescence or depletion-like phenotype (8–10). The degree of T-cell subset abnormality correlates with the severity of the disease and the outcome of the treatment (9, 10). T-cell dysfunction is not only affected by alterations in their phenotype, but mitochondrial dysfunction may also be involved (11).

T cell activation, proliferation, and differentiation are fundamentally driven by shifting in cellular metabolism, with mitochondria playing a central role (12). Mitochondria continuously remodel their mass, shape, size, and function by undergoing alternate processes of fission and fusion (13). Many reports have emphasized the importance of mitochondrial mass in controlling cell fate, including cell death, cell survival and cell differentiation (11, 14). Cells undergo mitochondrial remodeling and functional shift upon exposure to adverse stimuli (15). As the driver of oxidative phosphorylation (OXPHOS), ATP production and oxygen consumption, alterations in mitochondrial membrane potential (MMP) also lead to mitochondrial dysfunction triggering a range of cellular injuries. Previous studies have confirmed the presence of excessive oxidative stress and impaired energy metabolism in the T cells of depressed patients, and that immune abnormalities are closely associated with clinical outcomes (8, 16, 17). Due to limitations in testing technology, mature mitochondrial function assays have not yet been developed for clinical practice, and there are relatively few longitudinal studies of the immunometabolic status of hospitalized depressed patients.

Recently, a new flow cytometric probe technology has emerged, which employs a special fluorescent dye to specifically bind to mitochondria in living cells, and utilizes the median fluorescence intensity (MFI) to reflect the MM and the ratio of the strength of the MFI to reflect the MMP as detected by the probe technology, which is expressed here as the low mitochondrial membrane potential (MMP^Low^, %) (18–20). According to the measurement of MM and MMP^Low^ of immune cells, the mitochondrial fusion and division, energy metabolism, and the degree of damage and destruction can be reflected. In this study, T lymphocyte subpopulations were labeled with specific antibodies CD62L and CD45RA two labeled tetrads to label their subpopulations and to detect the percentage of naïve T (Tn), effector T (Tef), effector memory T (Tem), and central memory T (Tcm) cells (21), and to use this technique for the preliminary exploration of immune cell mitochondrial alterations in patients with depression (22). We used this technique to detect peripheral blood T-lymphocyte subpopulation ratios, MM and MMP^Low^ alterations in depressed patients in response to the mitochondrial energy metabolism of immune cells, and to exploit the value of MM and MMP^Low^ for clinical applications.

## 2 Methods

### 2.1 Study subjects

**Figure 1:**
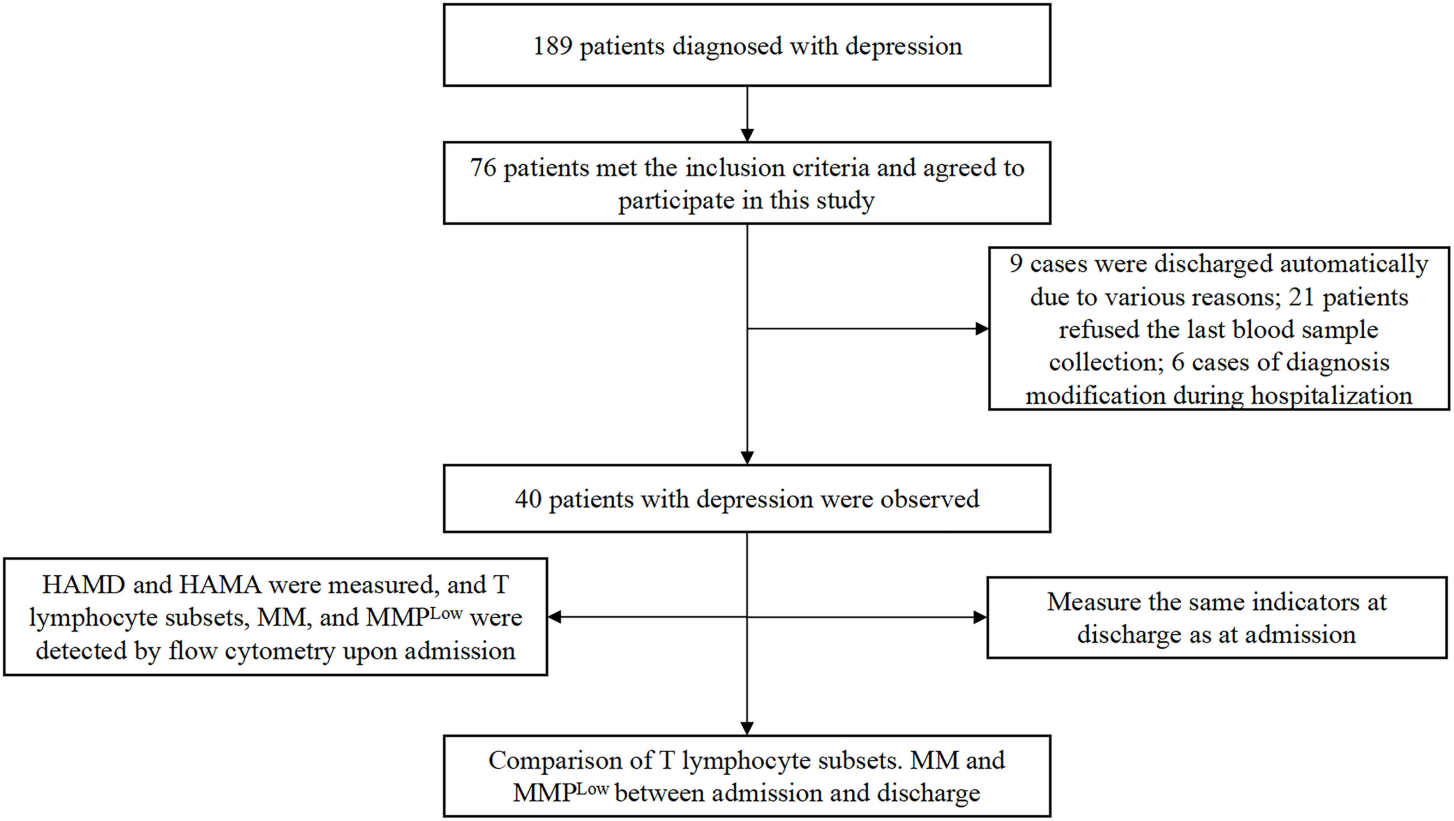
Flow chart for the study. HAMD, Hamilton Depression Scale (39); HAMA, Hamilton Anxiety Scale (40).

From January to June 2024, we obtained 40 depression inpatients from the Second People’s Hospital of Hunan Province. The study case screening process is shown in **Figure 1**. Inclusion criteria: 1) meeting the clinical diagnostic criteria of depression (International Classification of Diseases, ICD-10) (23); 2) not using immunomodulators in the last 1 year; 3) no infectious diseases in the last 2 weeks; 4) patients or family members giving informed consent to this study and signing the informed consent form; 5) aged 16–50 years old. Exclusion criteria: 1) comorbidity with other psychiatric conditions; 2) comorbidity with severe cardiac, hepatic, renal, pulmonary dysfunction or somatic diseases; 3) pregnant or lactating females; 4) alcohol/drug dependence; 5) obese patients with a BMI >28 kg/m^2^. The Second People’s Hospital of Hunan Province ethics committee approved the study, and all participants obtained informed written consent (Approval No. 2024K013).

### 2.2 T Lymphocyte subsets and mitochondrial indicators measurements

CD4+, CD8+ T-cell subsets (CD62L+CD45RA+ naïve T-lymphocytes, CD62L+CD45RA- central memory T-lymphocytes, CD62L-CD45RA+ effector T-lymphocytes, CD62L-CD45RA- effector memory T-lymphocytes were assayed using the DiagCyto 6C2L cytometric assay (UBBio technology, Hangzhou, China) (UB105441, UBBio Technology Co.) Procedure: 100 μL of peripheral whole blood sample with EDTA anticoagulant was mixed with antibodies (including CD4-FITC, CD8-APC-Cyanine7, CD45RA-PerCP-Cy5.5, CD62L-PE-Cyanine7) and incubated for 15 min at room temperature in the dark. 2 mL of hemolysin destroyed the red blood cells. The samples were then centrifuged at 300 x g for 5 min. The supernatant was discarded, and the precipitate was resuspended in 200 μL of PBS and transferred to an octuplicate tube containing MitoDye (C34H36Cl2N2, CN202110570964) and incubated at 37°C for 30 min with light protection. Finally, it was transferred to a flow tube and the labeled immune cells were counted by flow cytometry. NovoExpress software (Agilent Technology) was used for final analysis and graphical output. Side scatter area (SSC- H) versus cell viability marker plots were used to exclude dead cells. SSC-H and FSC-H plots help to identify lymphocyte and monocyte populations from granulocytes. The relative percentage of each lymphocyte subpopulation was measured, and the MM was calculated by combining the MFI of each subpopulation with the MFI of the lymphocyte mitochondrial function analysis system, and the ratio of the strength of the MFI was detected by the probe technology to respond to the MMP^Low^ (UBBio technology, Hangzhou, China, Patent No. CN202210495602.3).

### 2.3 Data presentation and statistical analysis

SPSS 25.0 and Prism 8.2.1 were used for data analysis. Outlier analysis was performed using Tukey’s method prior to statistical testing. The normality of data distribution was determined using the D’Agostino-Pearson test. Continuous variables were expressed as mean ± standard deviation or median (interquartile spacing), and paired data were compared using the Paired test or Wilcoxon T-test. All statistical tests were 2-tailed. Differences were considered statistically significant if *P*<0.05.

## 3 Results

### 3.1 Demographic and clinical characteristics

40 patients were finally included in the study, all of whom were diagnosed with depression and willing to undergo inpatient treatment. We defined depressed patients discharged within 21 days of hospitalization as having received more effective treatment, and defined patients treated for less than or equal to 21 days as the routine-term hospitalization group, and patients treated for more than 21 days as the long-term hospitalization group. We compared the clinical characteristics of the two groups (**Table 1**). The two groups showed no significant differences in baseline demographic or clinical features, including age, sex distribution, age at onset, and precipitating factors. Most of the patients had an unknown cause of onset, and stress and emotional problems were also the main causes of onset. There were no statistically significant differences in the use of atypical antipsychotics, benzodiazepines, mood stabilizers, NDRI, NaSSA, SNRI, SSRI, TCA or combination regimens between the two groups, which indicates that the distribution of psychotropic drug use between the two groups is generally balanced. Assessment scores at admission, including the HAMD and HAMA, were comparable between groups suggesting similar initial symptom severity. Only the length of hospital stays differed significantly between groups (*P* < 0.0001).

**Table 1:** Characteristics of patients.

Characteristics	Total patients (n=40)	Routine-term hospitalization (n=26)	Long-term hospitalization (n=14)	*P*
Female, n	26	17	9	>0.9999
Age	23.00 (13.75)	21.50 (10.50)	27.00 (19.50)	0.2740
Age at onset of illness	18.00 (11.50)	16.50 (12.25)	20.00 (9.25)	0.3399
Length of hospital stay	17.00 (8.00)	14.00 (6.00)	21.00 (5.25)	**<0.0001**
Inducing factors, n				0.4221
• Cause unknown	16	11	5	–
• Stress	12	7	5	–
• Emotional reasons	8	6	2	–
• Postpartum depression	1	0	1	–
• School bullying	1	0	1	–
• Physical reasons	2	2	0	–
Psychotropic drug use, n				0.4388
• Atypical antipsychotics	26	15	11	–
• Benzodiazepines	20	15	5	–
• Mood Stabilizers	11	8	3	–
• NDRI	2	1	1	–
• NaSSA	8	4	4	–
• SNRI	9	7	2	–
• SSRI	22	13	9	–
• TCA	2	0	2	–
Treating with two or more drugs	36	23	13	>0.9999
Admission scale score				
• HAMD	17.11 ± 7.27	17.83 ± 7.83	15.86 ± 6.27	0.4267
• HAMA	17.35 ± 8.67	17.88 ± 9.80	16.36 ± 6.26	0.6016

NDRI, norepinephrine-dopamine reuptake inhibitor; NaSSA, noradrenergic and specific serotonergic antidepressant; SNRI, serotonin-norepinephrine reuptake inhibitor; SSRI, selective serotonin reuptake inhibitor; TCA, tricyclic antidepressant.

*P*, Routine-term hospitalization group vs Long-term hospitalization group.

Significance is indicated by bold marking when *P*<0.05.

### 3.2 Changes in the percentage of T-cell subsets before and after treatment in patients with depression

**Figure 2:**
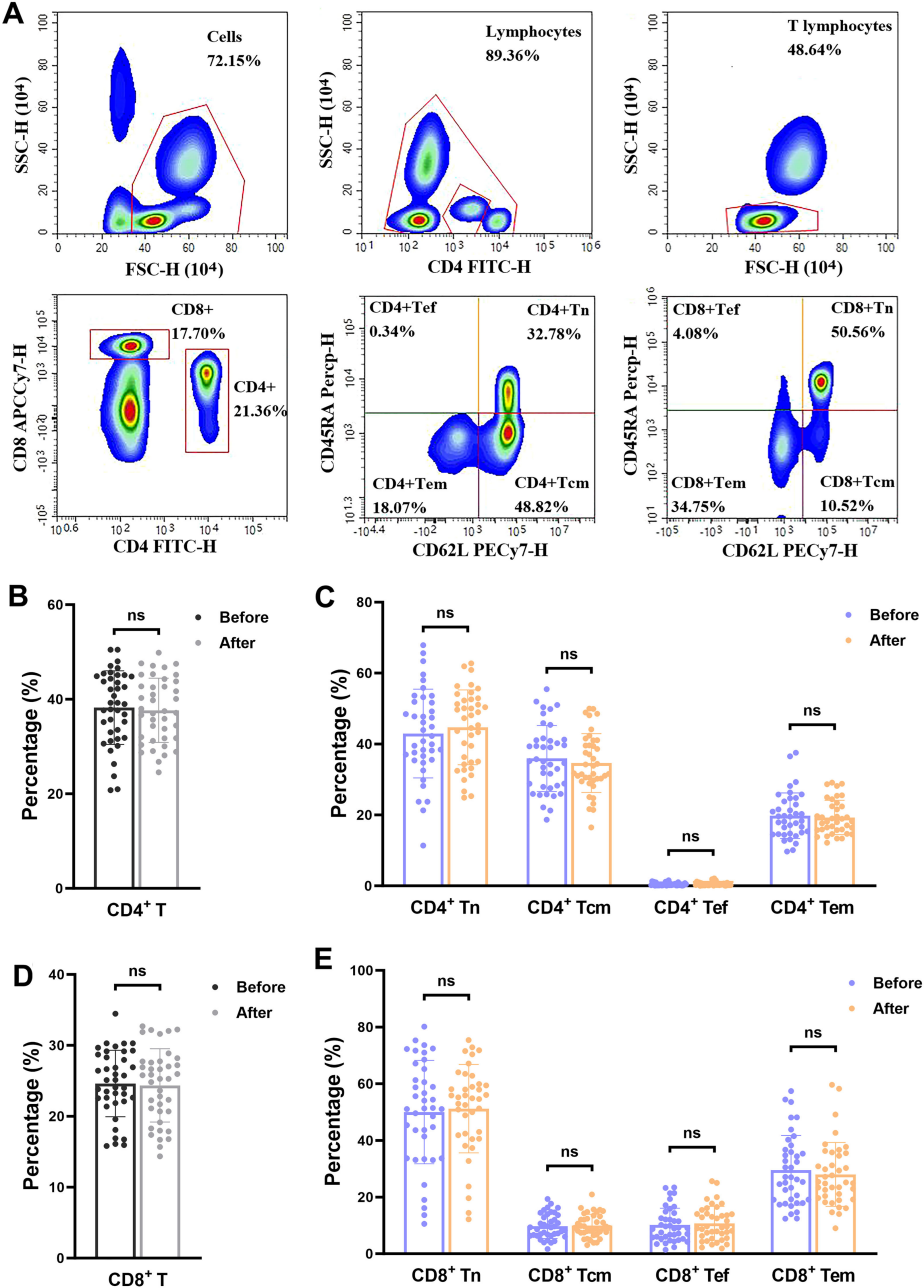
Percentage of T-cell subsets in depressed patients before and after treatment. **(A)** flow cytometry circle-gate rule. **(B-E)** percentage of T-cell subsets. ns, *P* > 0.05.

**Figure 3:**
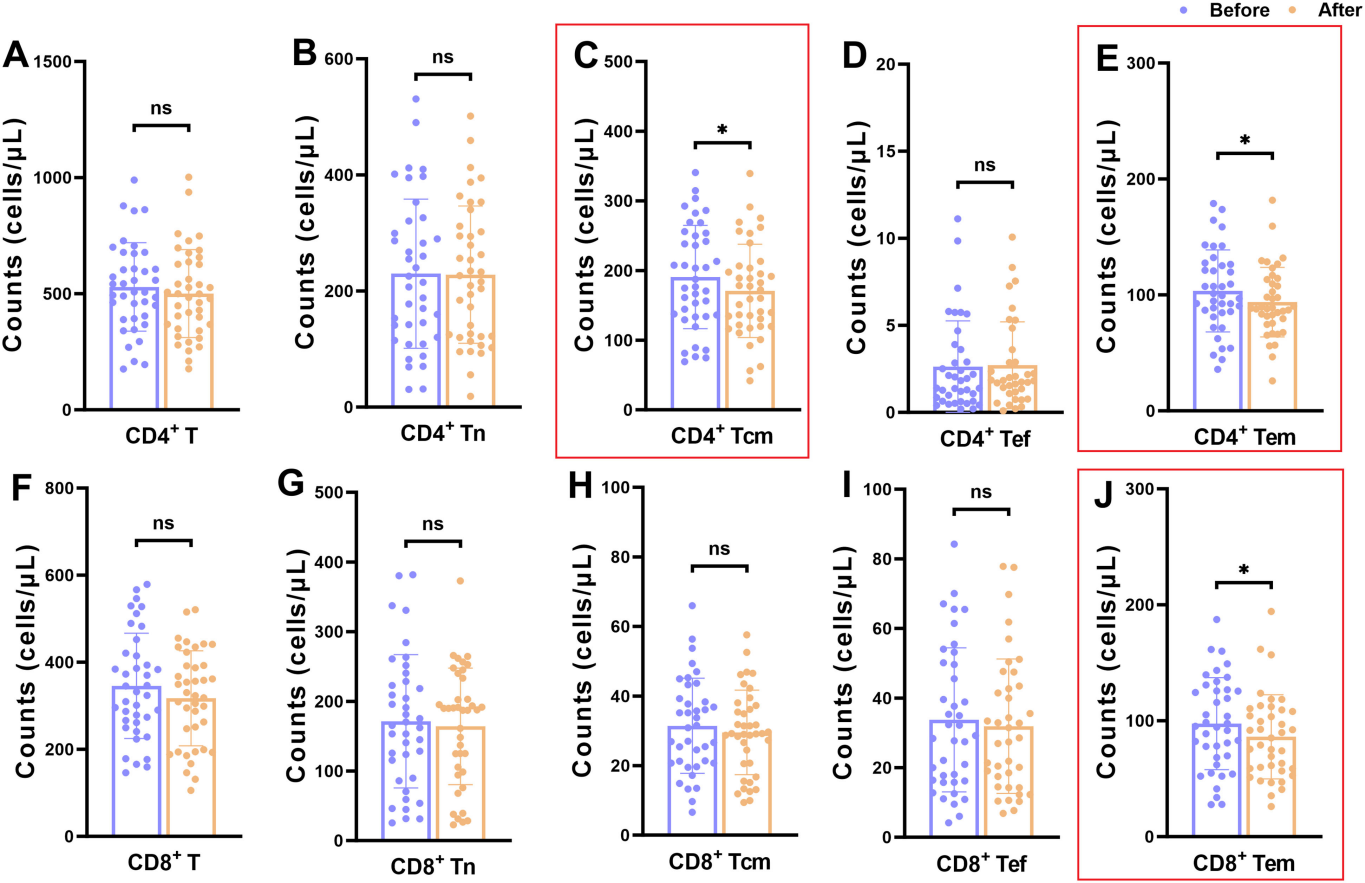
Counts of T-cell subsets in depressed patients before and after treatment. **(A-E)** Changes in counts of CD4+T cell subpopulations in depressed patients before and after treatment; **(F-J)** Changes in counts of CD8+T cell subpopulations in depressed patients before and after treatment. *P < 0.05; ns *P* > 0.05.

We analyzed differences in the counts and percentages of T lymphocyte subsets in depressed patients before and after treatment, including the total CD4+T and CD8+T cell populations and their subsets CD4+Tn, CD4+Tcm, CD4+Tef, CD4+Tem, CD8+Tn, CD8+Tcm, CD8+Tef, and CD8+Tem (**Figures 2**, **3**). Flow cytometry results demonstrated that there were no statistically significant differences in the frequencies of these T cell subsets after clinical treatment compared to baseline levels, indicating that the relative proportions of these T lymphocyte subsets remained stable throughout the treatment period (**Figure 2**). However, when comparing the absolute cell counts of each subset, we observed a significant decrease in the number of CD4+ Tcm, CD4+ Tem and CD8+ Tem after hospitalization (*P* < 0.05), while no significant changes were found in other subsets (**Figure 3**). This suggests that although the relative composition of T-cell subsets remained unchanged, specific memory subsets may have undergone quantitative contraction in response to treatment or hospitalization-related factors.

### 3.3 Changes in MM and MMP^Low^ of T-cell subsets before and after treatment in depression patients

**Figure 4:**
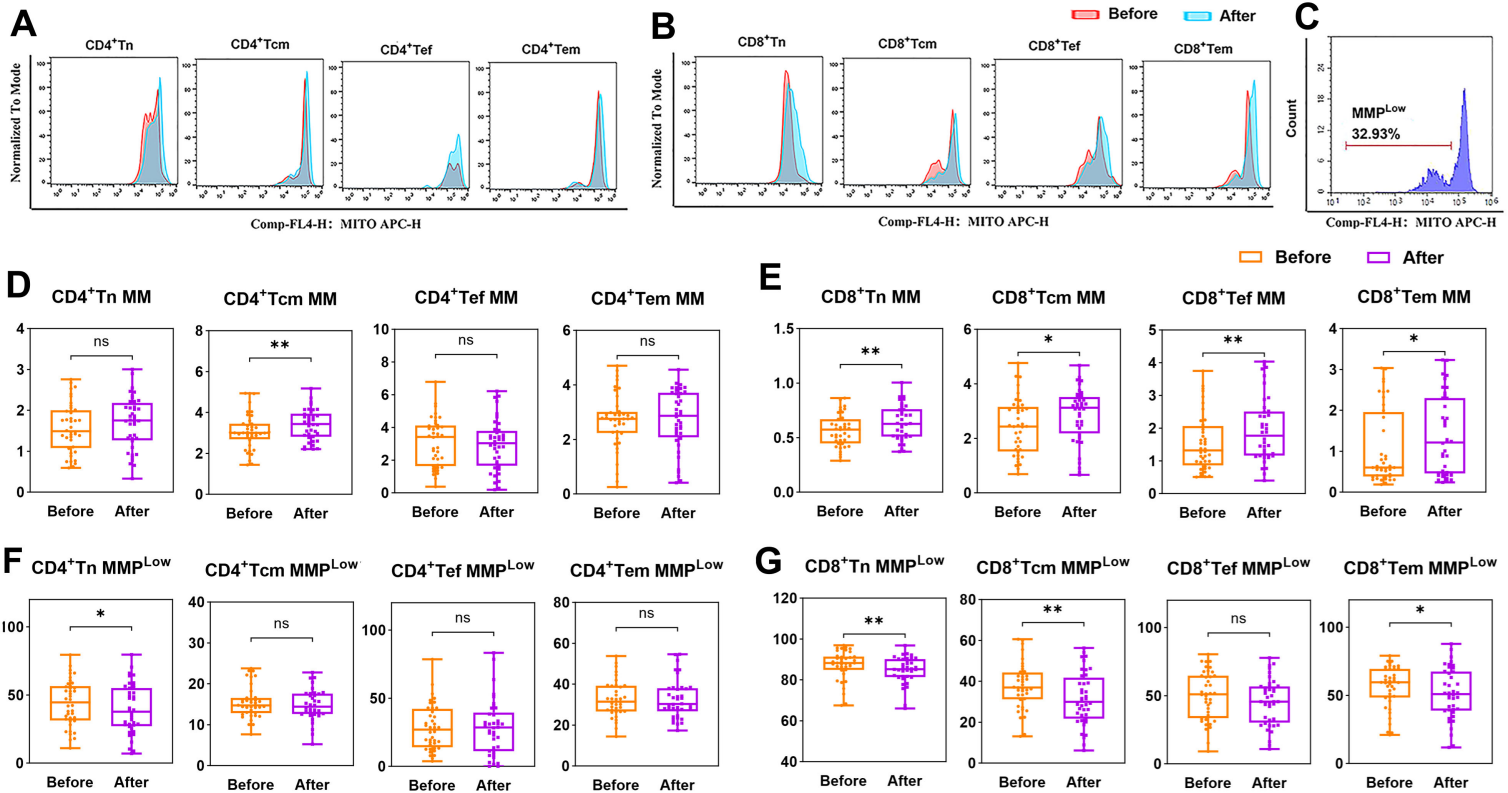
MM and MMP^Low^ of T-cell subsets in depressed patients before and after treatment. **(A-C)** Example of comparison of CD4+T and CD8+T cell subsets MM and MMP^Low^ before and after treatment. Mito/count histogram was set to distinguish the two groups of cells with low/high mitochondria. The median fluorescence intensity of the total group represented MM, and MMP^Low^ was obtained from the cell group with low APC fluorescence intensity. **(D, E)** Changes in MM of CD4+T and CD8+T cell subpopulations in depressed patients before and after treatment; **(F, G)** Changes in MMP^Low^ of CD4+T and CD8+T cell subpopulations in depressed patients before and after treatment. *P < 0.05; **P < 0.01; ns *P* > 0.05.

Mitochondrial metabolism is a key regulatory node driving T cell differentiation, and its dysfunction may participate in the pathological process of depression by altering immune response patterns (12, 24). The MM and MMP^Low^ of T-lymphocyte differentiated subpopulations in depressed patients(n=40) were analyzed and compared before and after treatment, including CD4+Tn, CD4+Tcm, CD4+Tef, CD4+Tem, CD8+Tn, CD8+Tcm, CD8+Tef, and CD8+Tem (**Figure 4**). Compared with baseline levels, except for CD4+ Tcm MM (*P* < 0.01), there were no statistically significant changes in MM of other CD4+ T cell subsets post-treatment (**Figure 4D**). Among the CD8+ T differentiated subsets, Tn, Tcm, Tef, and Tem were statistically different (*P* < 0.05), and all of them showed a rising trend after treatment (**Figure 4E**). After hospitalization, MMP^Low^ of CD4+Tn, CD8+Tn, CD8+Tcm and CD8+Tem cells was significantly reduced in depressed patients (*P* < 0.05) (**Figures 4F, G**). These reductions may indicate improved mitochondrial functionality and energy metabolism.

### 3.4 Outcome analysis of depressed patients discharged with a hospital stay of less than 21 days

**Figure 5:**
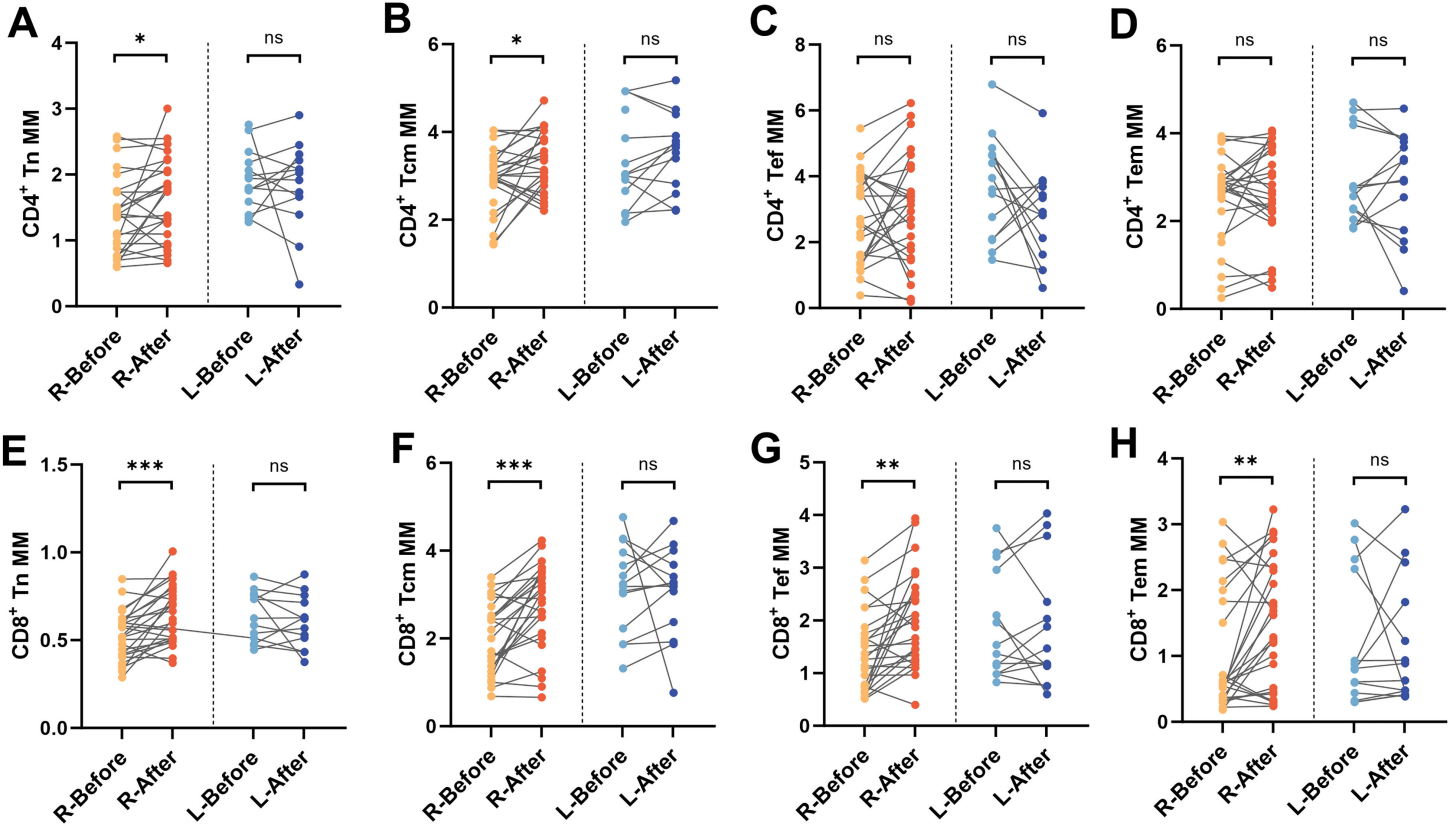
Changes in MM in depressed patients hospitalized for less than 21 days. Differences in MM of **(A)** CD4+Tn, **(B)** CD4+Tcm, **(C)** CD4+Tef, **(D)** CD4+Tem, **(E)** CD8+Tn, **(F)** CD8+Tcm, **(G)** CD8+Tef, and **(H)** CD8+Tem before and after treatment in routine-term hospitalization and long-term hospitalization group groups. R, Routine-term hospitalization group; L, Long-term hospitalization group, *P < 0.05; **P < 0.01; ***P < 0.001; ns *P* > 0.05.

We stratified patients into two subgroups based on hospitalization duration (≤21 days as routine-term, and >21 days as long-term hospitalization) to facilitate exploratory analysis of potential associations between immunometabolic responses and clinical treatment outcomes. In the long-term hospitalization group, no statistically significant changes in MM were observed in any of the CD4^+^ or CD8^+^ T-cell subsets. In contrast, the routine-term hospitalization group demonstrated significant post-treatment increases in MM within the CD4+ Tn (*P* < 0.05), CD4+ Tcm (*P* < 0.05), CD8+ Tn (*P* < 0.001), CD8+ Tcm (*P* < 0.001), CD8+ Tef (*P* < 0.01), and CD8+ Tem (*P* < 0.01) subsets (**Figure 5**). The observed increase in MM among specific T-cell subsets in the routine-term hospitalization group may preliminarily reflect a potential association between more rapid clinical improvement and favorable immune-metabolic adaptations.

## 4 Discussion

Activation of the immune system and changes in immune cells in patients with depression have been reported in the literature, but there is no definitive determination of the severity of immune damage, and few studies have been done on changes in mitochondrial energy metabolism and damage to immune cells in particular (19, 22, 25). In this study, the dynamic observation of the frequency and mitochondrial function of peripheral T-lymphocyte subpopulations in hospitalized depressed patients was used to explore immune cell damage and depletion during the course of the disease. Consistent with previous reports, the tendency toward terminal differentiation of CD4+/CD8+ T subpopulations in depressed patients was reversed after treatment (8–10). In exploring the alterations in mitochondrial MM and MMP^Low^ of peripheral T-lymphocytes in depressed patients and their relationship to disease regression, it was found that T-lymphocyte MM and MMP^Low^ appeared to be significantly altered in before and after treatment of the depressed patients, which suggests that the mitochondria of the immune cells of the depressed patients undergo observable microscopic alterations, even though the numbers and proportions of the immune cells are not significantly altered. Inconsistently, mitochondrial abnormalities in patients with self-immunization, infections, or tumors are accompanied by significant alterations in both lymphocyte numbers and proportions (26–28). This brings up two questions: first, whether changes in mitochondrial energy metabolism in immune cells necessarily lead to hypoplasia and apoptosis, and second, what exactly are the initiating factors (antigens) that stimulate immune responses in depressed patients.

**Figure 6:**
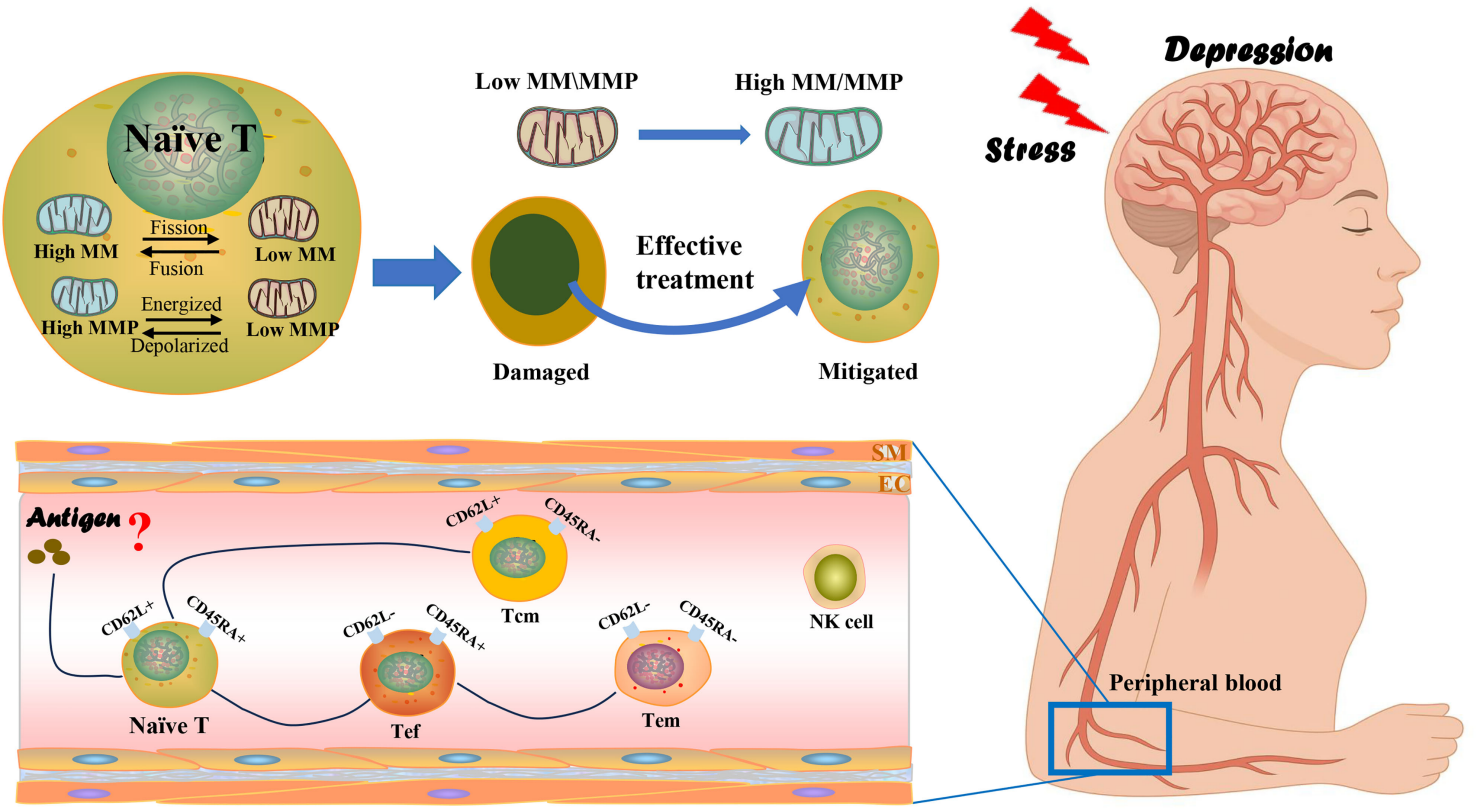
Mechanisms associated with T-lymphocyte subsets and mitochondrial MM and MMP in patients with depression. Stress-induced mitochondrial dysfunction in peripheral T lymphocyte subsets contributes to immune alterations in depression. Chronic stress in depressed patients disrupts MM and MMP within distinct CD4+ and CD8+ T cell subsets, impairing cellular metabolism and activation. Restoration of mitochondrial function in these subsets through effective antidepressant therapies may alleviate depressive symptoms by normalizing immune responses. Conversely, persistent mitochondrial dysfunction potentially sustains chronic inflammation, reinforcing depressive states.

As shown in **Figure 6**, theoretically, naive T cells receive antigenic stimulation and activate to become short-term acting effector T cells and long-term acting memory T cells. However, our study did not find any significant change in the percentage of T cell subsets before and after treatment of the depressed patients, i.e., no significant activation of the immune system was observed. This may be related to the small number of cases included in the study. Also, it is more likely that the pathogenesis of patients with depression is intertwined with complex factors, and the immune response presents different types and degrees depending on the pathogenic triggers, leading to the inability to present obvious trends in the overall analysis, which also puts a higher demand on depression disease typing.

Mitochondria, as bioenergetic organelles, fulfill most of the energy requirements of organisms by providing ATP. The metabolism of glucose, lipids, and proteins is connected through the tricarboxylic acid cycle and oxidative phosphorylation, which is the main function of mitochondria (29). In healthy individuals, different immune cell subpopulations have different levels of energy metabolism (30), which can be reflected by MM as well as MMP (30–32). During respiration and oxidation, mitochondria store the energy generated in the form of electrochemical potential energy in the inner membrane, causing the asymmetric distribution of protons and other ions on both sides of the inner membrane and the formation of MMP. Normal MMP is a prerequisite for mitochondrial oxidative phosphorylation and ATP production, and the stabilization of MMP is conducive to maintaining normal physiological functions of cells.

As shown in **Figure 6**, our study showed that the mitochondrial energy metabolism level of peripheral blood T-lymphocytes of depressed patients who received effective treatment showed significant changes, and exactly, effective treatment was helpful for the recovery of mitochondrial function. Fan et al. found that stress induces mitochondrial fission of CD4+ T cells in animals, leading to the overproduction of xanthine. The xanthine enters the brain via the bloodstream and directly stimulate the amygdala, causing anxiety-related behaviors, which is primarily caused by mitochondrial fission in CD4+ Tn cells (33). It is also in line with our finding that in Routine-term hospitalization group, pre-treatment depression patients’ CD4+Tn MM was lower than at discharge. Research also suggests that lymphocytes may cross the blood-brain barrier in inflammatory diseases to influence mood regulation and that cognition and social competence depend on a compassionate and fine-tuned balance of immune responses (22).

In the following patients, we found that depressed patients showed an overall trend of decreasing MMP^Low^ results for each CD8+ T cell subset during treatment, which did not seem to correlate directly with actual efficacy, but only with length of hospitalization. The reason for this analysis may be because some psychotropic drugs contain antioxidant components, such as N-acetylcysteine and coenzyme Q10, which directly improve mitochondrial activity (34), and olanzapine or clozapine, which may also protect mitochondrial activity through anti-inflammatory and antioxidant effects or modulation of mitochondrial gene expression (35). Since MMP is closely related to mitochondrial activity (30), most of the T-cell subpopulations showed a significant decrease in MMP^Low^ during the drug administration period. Although MM showed an upward trend after the treatment, it still showed a decrease in some of the patients, which means that there was a bifurcation in the change of MM. After grouping depression patients according to length of hospital stay, except for CD4+ Tn MM, CD4+ Tcm MM in the routine-term hospitalization group, there was no significant improvement in MM of other CD4+ T subpopulations before and after treatment. Whereas the trend of decline of CD8+ T-cell subsets MM before and after treatment was consistent and statistically different. In the long-term hospitalization group, there was no difference in either the MM of CD4+ or CD8+ T subgroups. It is well known that T cells are involved in the inflammatory response of the organism and promote or are directly involved in the immune response (36), and in the immune response of depressed patients, although no significant changes in the percentage of T cells were observed, which does not yet prove the existence of a stronger immune fluctuation in patients with depression, this microscopic change in the mitochondria of the immune cells that we observed could be a sign of a disease change related to the disease. We hypothesized that mitochondria in the immune cells of depressed patients play an important role in immune response and inflammatory reactions. It has been shown that the number and morphology of mitochondria in the immune cells of depressed patients are altered, with impaired mitochondrial function and reduced energy production. This mitochondrial damage may be a characteristic alteration in depressed patients. Cytokines such as TNF-α and IL-1β can affect the structure and function of mitochondria, leading to mitochondrial damage and impaired energy metabolism (37). Our results suggest that mitochondrial alterations in CD8+ T cells are associated with treatment responsiveness in depressed patients, and may reflect immune-metabolic changes that accompany the low-grade inflammatory state observed in depression.

Currently, there is a lack of quantitative diagnostic tools for psychosomatic disorders, and various psychosomatic disorders with different pathogenesis and treatment options are judged only based on clinical manifestations and scales. Whereas most mental illnesses are seen in primary health care, it is difficult to differentiate and diagnose different types of mental illnesses in the first instance. Considering the high prevalence of depression worldwide and the recognized consequences of untreated depression, the ability of primary care clinicians to treat and manage depression is critical (38), and objective, quantifiable laboratory test indices are the most effective tools for clinicians. Therefore, it is crucial to excavate depression-related laboratory test indicators that can be used clinically, and it is also an un-surmounted challenge to deeply excavate the pathogenesis of different types of psychiatric and psychological disorders from the perspective of immunology.

## 5 Conclusions

This study provides preliminary evidence of dynamic changes in T-cell MM and MMP^Low^ during the treatment of patients with depression. Notably, in patients with definite efficacy, alterations in T-cell subpopulation frequencies were minimal, whereas increases in MM and decreases in MMP^Low^ were more prominent, suggesting that mitochondrial indices may offer greater sensitivity for monitoring treatment response. These findings offer novel perspectives on the immunometabolic alterations of depression, and may contribute to advancing mechanistic understanding of its pathophysiology in future research.

## 6 Limitations

Several limitations merit consideration in this study. First, the absence of a healthy control group constrains the interpretation of whether the observed mitochondrial alterations are specific to depression or attributable to treatment effects or patient selection factors. Second, although MM and MMP^Low^ were assessed using flow cytometry, the lack of additional validation using structural mitochondrial markers (e.g., TOM20, VDAC1) and functional indicators (e.g., mitochondrial ROS) precludes deeper mechanistic insight. Third, assessment at only two time points (admission and discharge), dictated by technical constraints, hinders characterization of mitochondrial dynamics throughout the treatment course. Finally, the modest sample size and absence of stratification by medication regimen impede robust evaluation of potential drug effects, which warrants further investigation in larger cohorts.

## Data Availability

The raw data supporting the conclusions of this article will be made available by the authors, without undue reservation.
